# Case report: A novel *de novo* variant of *SCN8A* in a child with benign convulsions with mild gastroenteritis

**DOI:** 10.3389/fneur.2022.995513

**Published:** 2022-09-16

**Authors:** Hui Chen, Xiaoyan Li, Huaping Wu, Xiaolan Sun, Yuanyuan Che, Jian Zha, Ruiyan Wang, Xiongying Yu, Yong Chen, Jianmin Zhong

**Affiliations:** Department of Neurology, The Affiliated Children's Hospital of Nanchang University (Jiangxi Provincial Children's Hospital), Nanchang, China

**Keywords:** *SCN8A*, gene, benign, convulsions, gastroenteritis

## Abstract

Benign convulsions with mild gastroenteritis (CwG) is characterized by afebrile convulsions accompanied by mild gastroenteritis, and it can be considered after central nervous system infection, hypoglycemia, electrolyte disturbance, and moderate and severe dehydration are excluded. Previous studies have suggested that genetics may be involved in CWG. Herein, we reported a novel *de novo* variant of *SCN8A* in a child with CwG. This is the first report that *SCN8A* may be associated with CwG. Our report may provides evidence for the genetic etiology of CwG and expands the phenotypic and genetic spectrum of *SCN8A*-related disorders, which previously included severe developmental and epileptic encephalopathy (DEE) phenotype, benign epilepsy phenotype, spectrum of intermediate epilepsies, and patients with cognitive and/or behavioral disturbances without epilepsy. Phenotype of CwG has a good prognosis, and it does not require long-term antiepileptic therapy. Overtreatment should be avoided clinically. However, the conclusion needs to be further defined by long-term follow-up and similar clinical reports. In spite of this, our clinical observation provides possible evidence for future studies on the relationship between *SCN8A* and CwG.

## Introduction

Benign convulsions with mild gastroenteritis (CwG) was first reported by Morooka in 1982 in Japan (1), which was later repeatedly reported (2, 3). Most of the CwG cases occurs during the winter and early spring months. This clinical condition is characterized by afebrile convulsions accompanied by mild gastroenteritis in previously healthy infants. Besides, it can be considered after central nervous system infection, hypoglycemia, electrolyte disturbance, and moderate and severe dehydration are excluded, and usually has a good prognosis (4).

CwG has been more frequently described in East Asian countries, which suggests that the genetic characteristics of the host may play a role in the development of CwG (4). In addition to this, Okumura et al. found that CwG occurred in identical twins during the course of gastroenteritis, and their convulsions occurred almost simultaneously (5). These studies suggest that CwG may be related to genetic factors. However, in recent years, several scholars have tested *SCN1A, SCN1B*, and *PRRT2* genes in CwG patients, but no positive results were found (6–8). Finally, in 2020, Terrone et al. found heterozygous missense mutation of *SPTAN1* gene by next generation sequencing (NGS) in a family, including a 13-year-old sister, an 8-year-old brother and their 39-year-old mother (9). The clinical observation suggests for the first time that variants in *SPTAN1* gene might be involved in the etiology of CwG. Herein, we reported a novel *de novo* variant of *SCN8A* in a child with CwG. This is the first report that *SCN8A* may be associated with CwG. Our report may provides evidence for the genetic etiology of CwG and expands the phenotypic and genetic spectrum of *SCN8A*-related disorders.

## Case description

**Figure 1:**
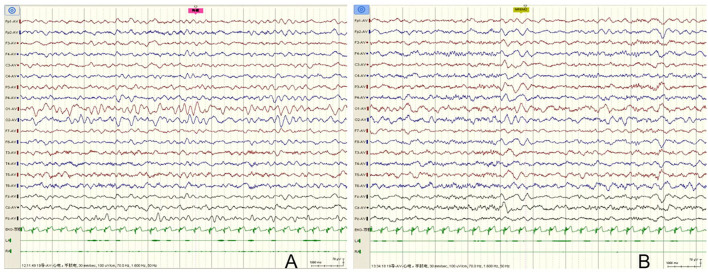
EEG of the case. **(A)** EEG showed slow background activity during wakefulness in interictal periods, and no epileptic discharges were observed. **(B)** No epileptic discharges were observed during sleep stages.

The patient was a 36 months old boy. The Chinese boy was born full term with normal birth weight. She had no history of transient hypoxia at birth. There was no family history of neurologic disorders. He developed acute gastroenteritis symptoms at 19 months, presenting with 3 times of vomiting and diarrhea (3 times daily). A day later, the boy began with 3 generalized tonic-clonic seizures (GTCS) without fever in 1 day. The duration of each convulsion was 2–3 min. Since then, phenobarbital was given (5 mg/kg, intravenous injection). However, he still had two more similar convulsions. After treatment of diazepam (0.3 mg/kg, intravenous injection), there was no further convulsion. Vomiting resolved within 2 days, while diarrhea recurred over 5 days (2–4 times a day) and then resolved. Dehydration did not occur throughout the course of the disease. In the interictal phase, the child's mental state was normal and his consciousness was clear. However, 10 days later, he developed symptoms of gastroenteritis again, including diarrhea and vomiting, and had one GTCS a day later. The duration of the convulsion was 2 min. The symptoms of the mild gastroenteritis lasted for 4 days, and the convulsions only occurred once during the course of the gastroenteritis. We did not give the child long-term antiepileptic treatment. After a long-term follow-up of 17 months, he never had another seizure. The child's motor and intellectual milestones were the same as those of normal children. Physical examination at the age of 19 months showed a height of 83 cm (50–85th centile), weight of 13.5 kg (85–97th centile) and head circumference of 47 cm (25–50th centile). His consciousness was clear. Cardiac auscultation was normal. Muscle strength and tone were normal. Knee reflex were normal and babinski signs were negative. EEG showed slow background activity during wakefulness in interictal periods, and no epileptic discharges were observed (Figure 1). Rotavirus of stool was positive. Other investigations were non-diagnostic including liver function tests, renal function, electrolytes, glucose, blood white cells, blood red cells, platelets, ammonia, creatine kinase, plasma lactate, white blood cells and red blood cells in stool, electrocardiogram, and brain MRI. Therefore, he was eventually diagnosed as suffering from CwG. Due to recurrent convulsions, the child's parents wanted to further understand the genetic causes, so we performed NGS on him. He was found to have a *de novo* heterozygous mutations c.5503 (exon27) C>G in *SCN8A* gene that was not detected in either parent. The variant led to protein changes p.Pro1835Ala (NM_014191.4). The variant (p.Pro1835Ala) was a *de novo* heterozygous variant (PS2), which affected highly conserved amino acid region. It was not found in the normal control population in multiple databases, such as gnomAD, ExAC, 1,000 Genomes, ESP6500 (PM2_Supporting). Various statistical methods predicted that the variation would have deleterious effects on gene products (PP3), such as SIFT (Damaging), MutationTaster (Disease_causing), FATHMM (Damaging), PROVEAN (Damaging), DANN (Damaging), CADD (Damaging) and Eigen (Damaging). In addition to this, the missense mutation of *SCN8A* gene had a high pathogenic possibility, and the Z-score was 7.64 in gnomAD database (PP2). Hence, the variant was classified as likely pathogenic in accordance with the ACMG guideline (PS2 + PM2_Supporting + PP2 + PP3).

## Discussion

In our case report, the patient presented with multiple afebrile convulsions accompanied by mild gastroenteritis, and he did not present with electrolyte disturbances, dehydration, hypoglycemia, or central nervous system infection. In addition, he did not receive long-term antiepileptic medication. After a long-term follow-up of 17 months, he did not develop convulsions again, and his motor and cognitive development was normal. he has a good prognosis. In the first course of acute gastroenteritis, vomiting resolved within 2 days, while diarrhea resolved within 5 days. Ten days later, he developed symptoms of gastroenteritis again, including diarrhea and vomiting. The symptoms of the mild gastroenteritis lasted for 4 days. The convulsions only occurred during the course of the gastroenteritis. During a 10-day period, there were no symptoms of gastroenteritis or convulsions. Therefore, I think it was two episodes. Recent study has shown that children with CwG have a possibility of recurrence, with a recurrence rate of about 6.3% (10). Therefore, child of the case might suffer from recurrent CwG according to its diagnostic criteria (4, 10). However, because the interval time is too short (10-day period), it needs to be differentiated from epilepsy. The diagnosis of recurrent CwG still needs to be further defined by long-term follow-up and similar clinical reports. CwG is similar to an epileptic syndrome within benign infantile seizures in the classification set by the International League Against Epilepsy (ILAE) because of its clinical features of afebrile convulsions. However, until now, CwG has not been fully recognized as an epileptic syndrome by ILAE. Because convulsion of CwG is accompanied by mild gastroenteritis, it has been suggested that CwG might be termed as situation-related seizures (4). In conclusion, CwG is a separate disease which is different from epilepsy.

*SCN8A* encodes Nav1.6, which is one of four voltage-gated sodium channels expressed in the mammalian brain. Nav1.6 is found in the central and peripheral nervous system with a predominant expression in excitatory, but also in inhibitory neurons (11, 12). The first pathogenic variants in *SCN8A* have been described in an affected individual with developmental and epileptic encephalopathy (DEE) (13). In recent years, a wide clinical spectrum of neurodevelopmental phenotypes has been reported, including severe DEE phenotype, benign epilepsy phenotype, spectrum of intermediate epilepsies, generalized epilepsy, unclassifiable epilepsy and patients with cognitive and/or behavioral disturbances without epilepsy (12, 14–17). Most pathogenic variants in SCN8A are missense. Previous functional studies of selected epilepsy-associated *SCN8A* variants have revealed a gain of function (GoF) pathogenic mechanism, which cause ultimately hyperactivity of the ion channel. On the contrary, variants causing loss of function (LoF) are related with patients with cognitive and/or behavioral disturbances without epilepsy, Such as ID, ASD, myoclonus, and ataxia (14). However, this view is no longer true, a recent study showed generalized epilepsy with absence seizures was the main epilepsy phenotype of LOF variant carriers and the extent of the electrophysiological dysfunction of the GOF variants was a main determinant of the severity of the clinical phenotype in focal epilepsies including severe DEE phenotype, benign epilepsy phenotype, spectrum of intermediate epilepsies. However, a few functional studies of *SCN8A* variants in DEE were LOF (12). Our clinical observation is the first report that *SCN8A* may be associated with CwG. Our clinical observation is only a case report, which need to be supported by more case reports. Moreover, relevant functional studies will be needed to further clarify the related mechanisms. This is the limitation of our clinical observation.

In conclusion, our clinical observation suggests that variants in *SCN8A* gene might be involved in pathogenesis of CwG. Phenotype of CwG has a good prognosis. It does not require long-term antiepileptic therapy, and overtreatment should be avoided clinically. However, the conclusion needs to be further defined by long-term follow-up and similar clinical reports. In spite of this, our clinical observation provides possible evidence for future studies on the relationship between *SCN8A* and CwG.

## Data availability statement

The raw data supporting the conclusions of this article will be made available by the authors, without undue reservation.

## Ethics statement

The studies involving human participants were reviewed and approved by the Research Ethics Committee of Children's Hospital of Jiangxi Province. Written informed consent to participate in this study was provided by the participants' legal guardian/next of kin. Written informed consent was obtained from the individual(s), and minor(s)' legal guardian/next of kin, for the publication of any potentially identifiable images or data included in this article.

## Author contributions

All authors listed have made a substantial, direct, and intellectual contribution to the work and approved it for publication.

## Conflict of interest

The authors declare that the research was conducted in the absence of any commercial or financial relationships that could be construed as a potential conflict of interest.

## Publisher's note

All claims expressed in this article are solely those of the authors and do not necessarily represent those of their affiliated organizations, or those of the publisher, the editors and the reviewers. Any product that may be evaluated in this article, or claim that may be made by its manufacturer, is not guaranteed or endorsed by the publisher.
